# Supplementary material for: Satellite-derived temperature measures miss key physiologically relevant thermal trends on Palauan reefs
Source: PLoS One. 2026 Feb 2;21(2):e0341926. doi: 10.1371/journal.pone.0341926 (PMC12863525; doi:10.1371/journal.pone.0341926)
Supplement: S1 Table — Bonferroni correction applied for a family of 4 tests. Newey-West/Beck & Katz correction applied to regional/full SEs. (PDF) [file pone.0341926.s001.pdf]

Region	Parameter	Estimate	Standard Error	T-value	DoF	P-value	Cor	Mean_Difference (SSST-InSitu)	Metric
all	(Intercept)	-0.489	0.414	-1.181	2515	0.951	0.895	-0.578	Mean_Temp
all	SSST	1.036	0.014	2.58	2515	0.04	0.895	-0.578	Mean_Temp
Northern	(Intercept)	-1.564	1.529	-1.023	644	1	0.887	-0.392	Mean_Temp
Northern	SSST	1.067	0.053	1.271	644	0.817	0.887	-0.392	Mean_Temp
Southern	(Intercept)	0.899	0.742	1.212	933	0.904	0.926	-0.575	Mean_Temp
Southern	SSST	0.989	0.025	-0.437	933	1	0.926	-0.575	Mean_Temp
Western	(Intercept)	0.388	1.198	0.324	934	1	0.896	-0.708	Mean_Temp
Western	SSST	1.011	0.041	0.268	934	1	0.896	-0.708	Mean_Temp
all	(Intercept)	-0.517	0.597	-0.867	2515	1	0.814	-1.003	Max_Temp
all	SSST	1.052	0.02	2.552	2515	0.043	0.814	-1.003	Max_Temp
Northern	(Intercept)	-4.634	2.884	-1.607	644	0.434	0.782	-0.999	Max_Temp
Northern	SSST	1.193	0.099	1.936	644	0.213	0.782	-0.999	Max_Temp
Southern	(Intercept)	1.066	0.939	1.135	933	1	0.897	-0.908	Max_Temp
Southern	SSST	0.995	0.032	-0.168	933	1	0.897	-0.908	Max_Temp
Western	(Intercept)	0.263	1.864	0.141	934	1	0.798	-1.101	Max_Temp
Western	SSST	1.028	0.064	0.448	934	1	0.798	-1.101	Max_Temp
all	(Intercept)	-0.676	0.415	-1.627	2515	0.416	0.881	-0.316	Min_Temp
all	SSST	1.034	0.014	2.389	2515	0.068	0.881	-0.316	Min_Temp
Northern	(Intercept)	-1.521	1.486	-1.023	644	1	0.883	-0.077	Min_Temp
Northern	SSST	1.055	0.051	1.071	644	1	0.883	-0.077	Min_Temp
Southern	(Intercept)	0.957	0.852	1.124	933	1	0.904	-0.308	Min_Temp
Southern	SSST	0.978	0.029	-0.759	933	1	0.904	-0.308	Min_Temp
Western	(Intercept)	0.402	1.065	0.378	934	1	0.897	-0.488	Min_Temp
Western	SSST	1.003	0.036	0.081	934	1	0.897	-0.488	Min_Temp
all	(Intercept)	-1.619	1.514	-1.069	92	1	0.939	-0.588	Month_Mean
all	SSST	1.075	0.052	1.459	92	0.591	0.939	-0.588	Month_Mean
Northern	(Intercept)	-3.627	2.46	-1.474	22	0.618	0.934	-0.393	Month_Mean
Northern	SSST	1.138	0.087	1.59	22	0.504	0.934	-0.393	Month_Mean
Southern	(Intercept)	-0.093	1.052	-0.089	33	1	0.964	-0.591	Month_Mean
Southern	SSST	1.023	0.036	0.644	33	1	0.964	-0.591	Month_Mean
Western	(Intercept)	-0.268	1.351	-0.198	33	1	0.96	-0.72	Month_Mean
Western	SSST	1.034	0.047	0.723	33	1	0.96	-0.72	Month_Mean
all	(Intercept)	0.014	0.002	6.419	2515	< 0.001	0.724	-0.02	Over_BT
all	SSST	1.503	0.035	14.305	2515	< 0.001	0.724	-0.02	Over_BT
Northern	(Intercept)	0.003	0.003	1.301	644	0.775	0.798	-0.007	Over_BT
Northern	SSST	1.391	0.333	1.177	644	0.959	0.798	-0.007	Over_BT
Southern	(Intercept)	0.011	0.006	1.883	933	0.24	0.772	-0.012	Over_BT

Southern	SSST	1.069	0.036	1.91	933	0.226	0.772	-0.012	Over_BT
Western	(Intercept)	0.025	0.006	4.096	934	< 0.001	0.758	-0.036	Over_BT
Western	SSST	2.177	0.214	5.509	934	< 0.001	0.758	-0.036	Over_BT
all	(Intercept)	1.666	0.63	2.646	2512	0.033	-0.012	28.029	Diurnal_Var
all	SSST	-0.011	0.021	-47.248	2512	< 0.001	-0.012	28.029	Diurnal_Var
Northern	(Intercept)	-0.422	3.193	-0.132	643	1	0.069	27.338	Diurnal_Var
Northern	SSST	0.079	0.11	-8.372	643	< 0.001	0.069	27.338	Diurnal_Var
Southern	(Intercept)	0.263	1.038	0.253	932	1	0.057	28.308	Diurnal_Var
Southern	SSST	0.03	0.035	-27.436	932	< 0.001	0.057	28.308	Diurnal_Var
Western	(Intercept)	-0.9	1.641	-0.548	933	1	0.1	28.227	Diurnal_Var
Western	SSST	0.07	0.056	-16.569	933	< 0.001	0.1	28.227	Diurnal_Var
all	(Intercept)	0.103	0.124	0.829	289	0.408	0.978	-0.332	DHW
all	SSST	2.601	0.270	5.923	289	< 0.001	0.978	-0.332	DHW
all	(Intercept)	0.47	0.332	1.416	2512	0.628	0.924	-0.452	Daily_Avg
all	SSST	0.999	0.011	-0.054	2512	1	0.924	-0.452	Daily_Avg
Northern	(Intercept)	1.102	0.882	1.25	643	0.847	0.934	-0.282	Daily_Avg
Northern	SSST	0.972	0.03	-0.923	643	1	0.934	-0.282	Daily_Avg
Southern	(Intercept)	1.015	0.657	1.543	932	0.492	0.94	-0.461	Daily_Avg
Southern	SSST	0.981	0.022	-0.842	932	1	0.94	-0.461	Daily_Avg
Western	(Intercept)	0.973	0.843	1.155	933	0.994	0.925	-0.561	Daily_Avg
Western	SSST	0.986	0.029	-0.489	933	1	0.925	-0.561	Daily_Avg
