# Supplementary figures and images for: Satellite-derived temperature measures miss key physiologically relevant thermal trends on Palauan reefs
Source: PLoS One. 2026 Feb 2;21(2):e0341926. doi: 10.1371/journal.pone.0341926 (PMC12863525; doi:10.1371/journal.pone.0341926)

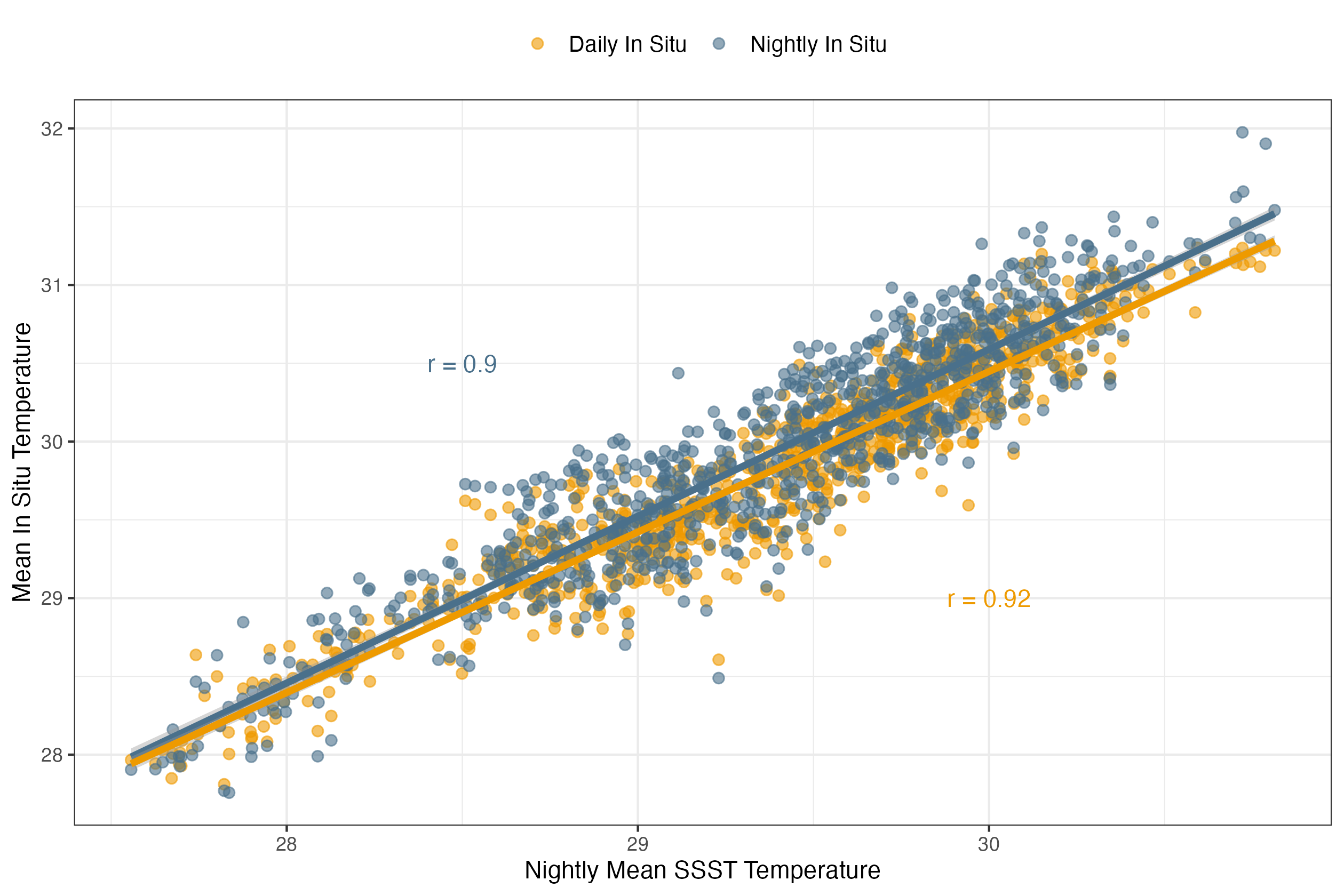

Supplement: S1 Fig — Colored lines are trendlines. Pearson correlation values are denoted in the color of the comparison. (TIF) [file pone.0341926.s002.tif]

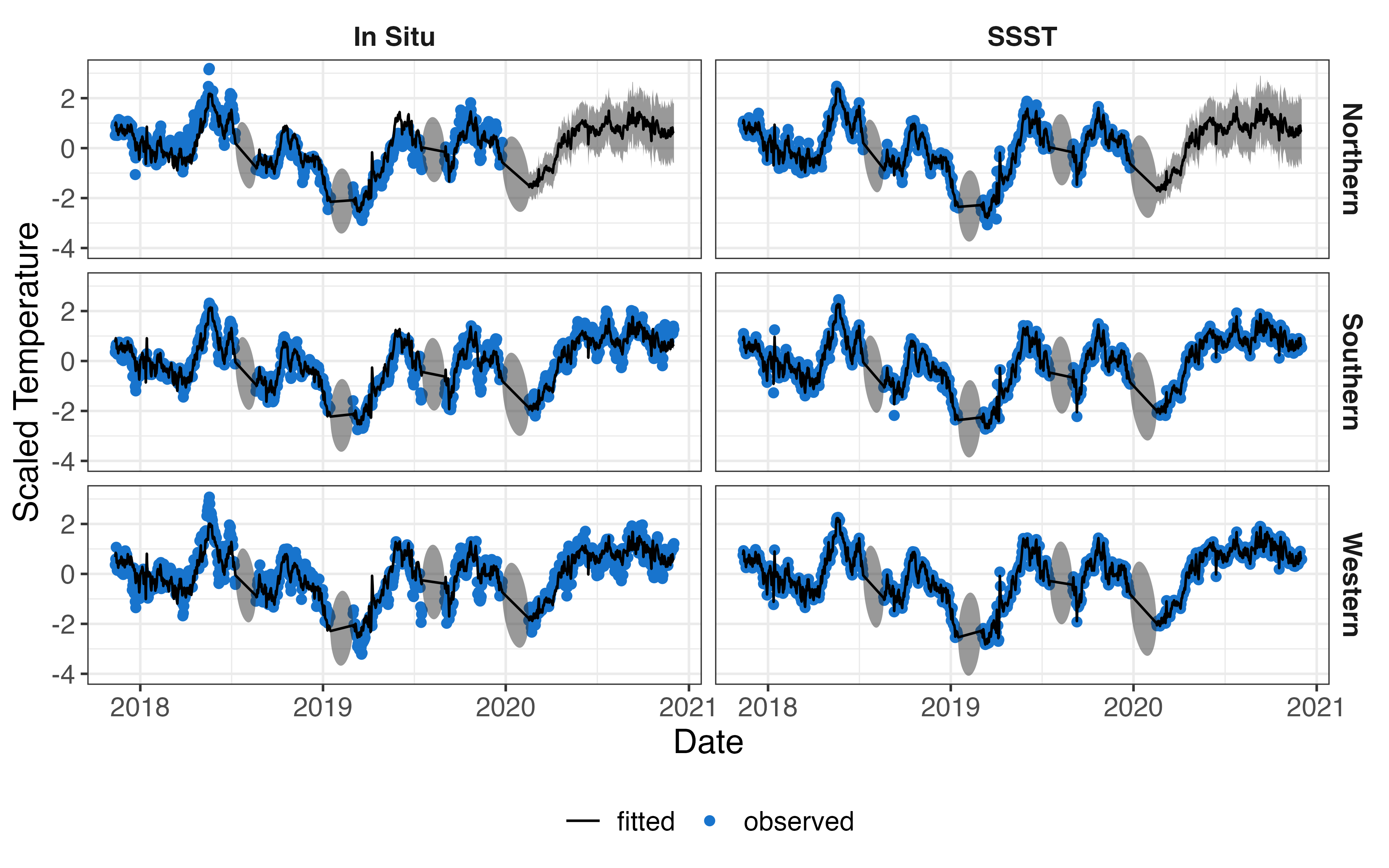

Supplement: S2 Fig — (TIF) [file pone.0341926.s003.tif]

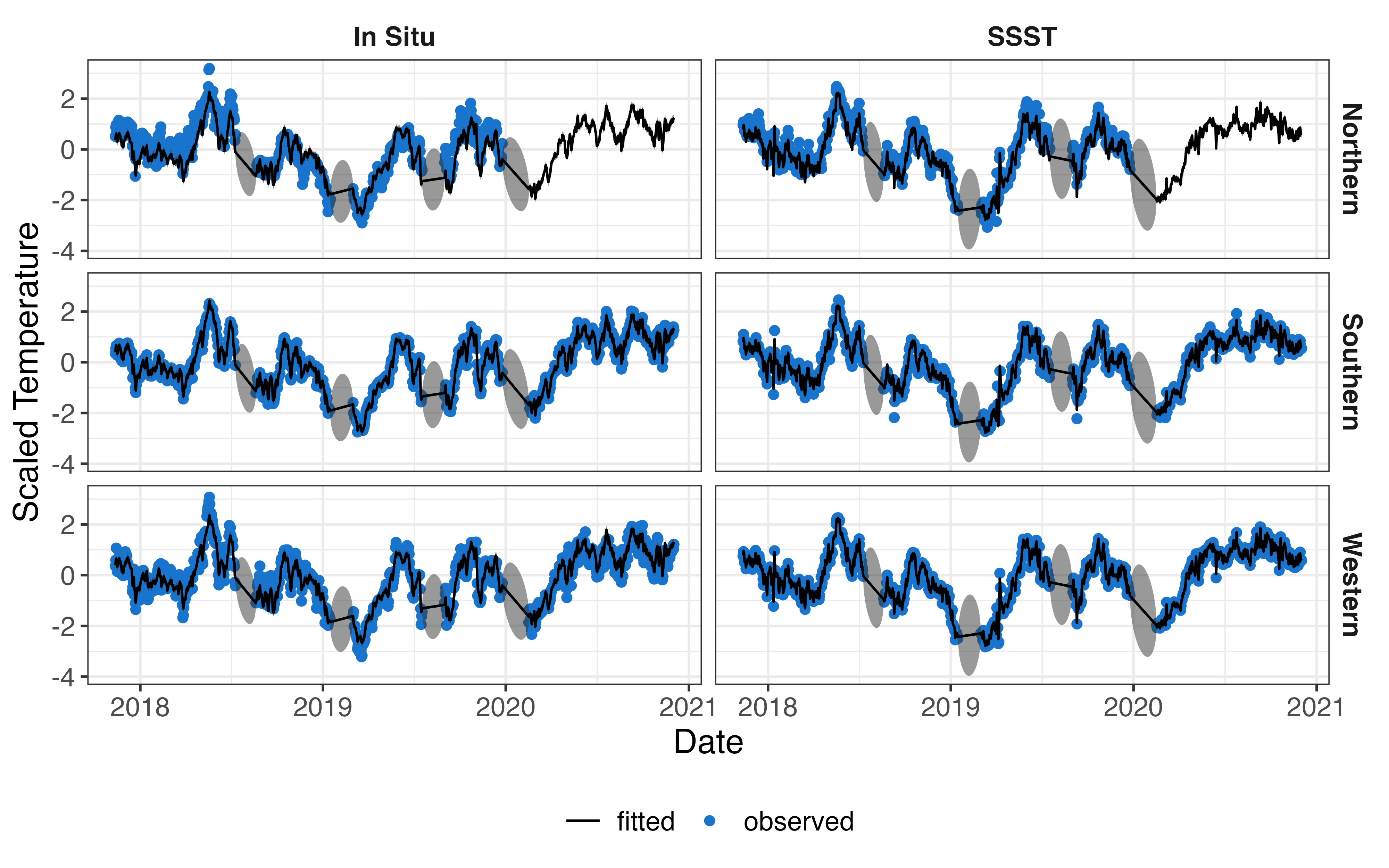

Supplement: S3 Fig — (TIF) [file pone.0341926.s004.tif]

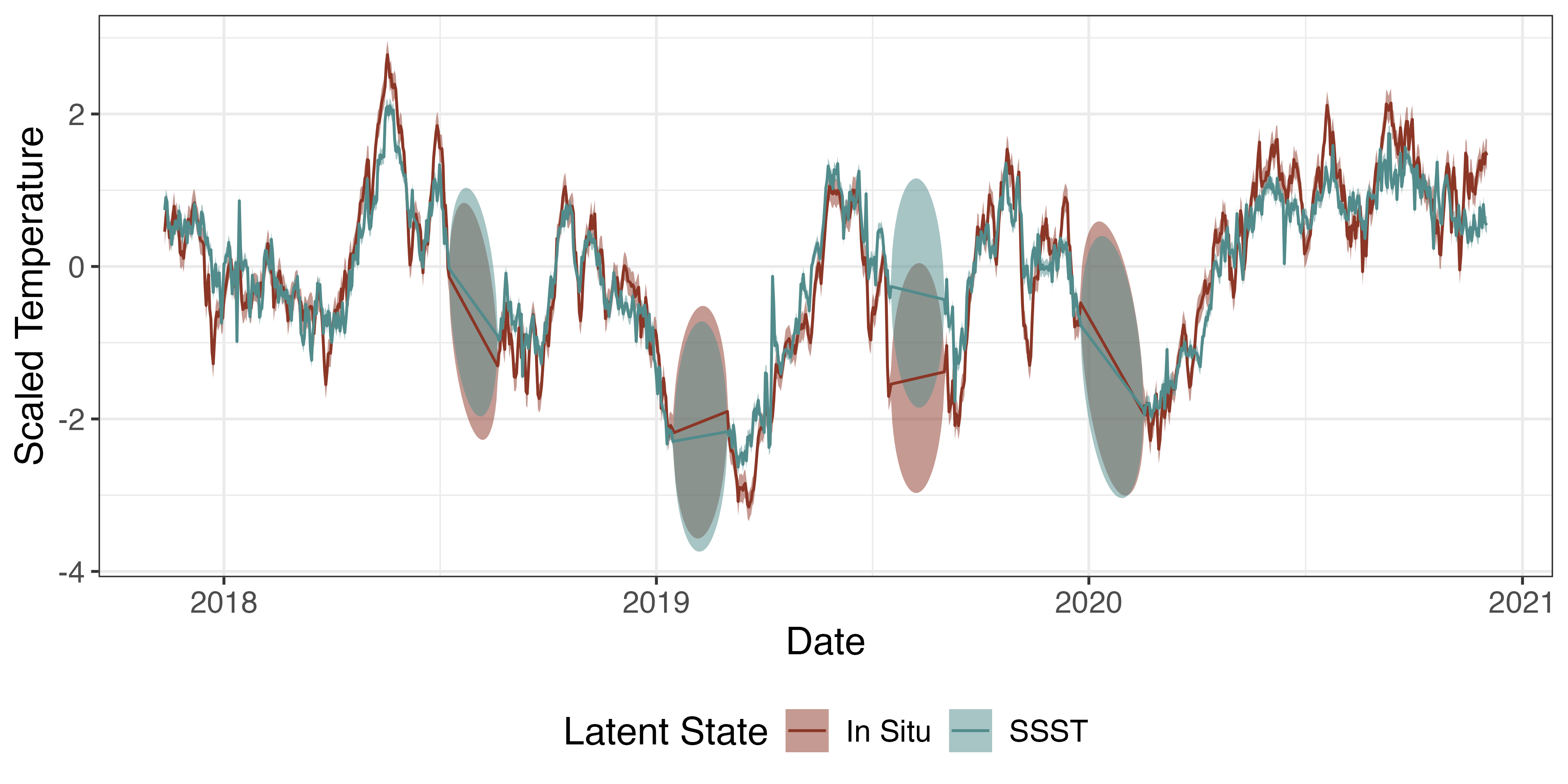

Supplement: S4 Fig — (TIF) [file pone.0341926.s005.tif]

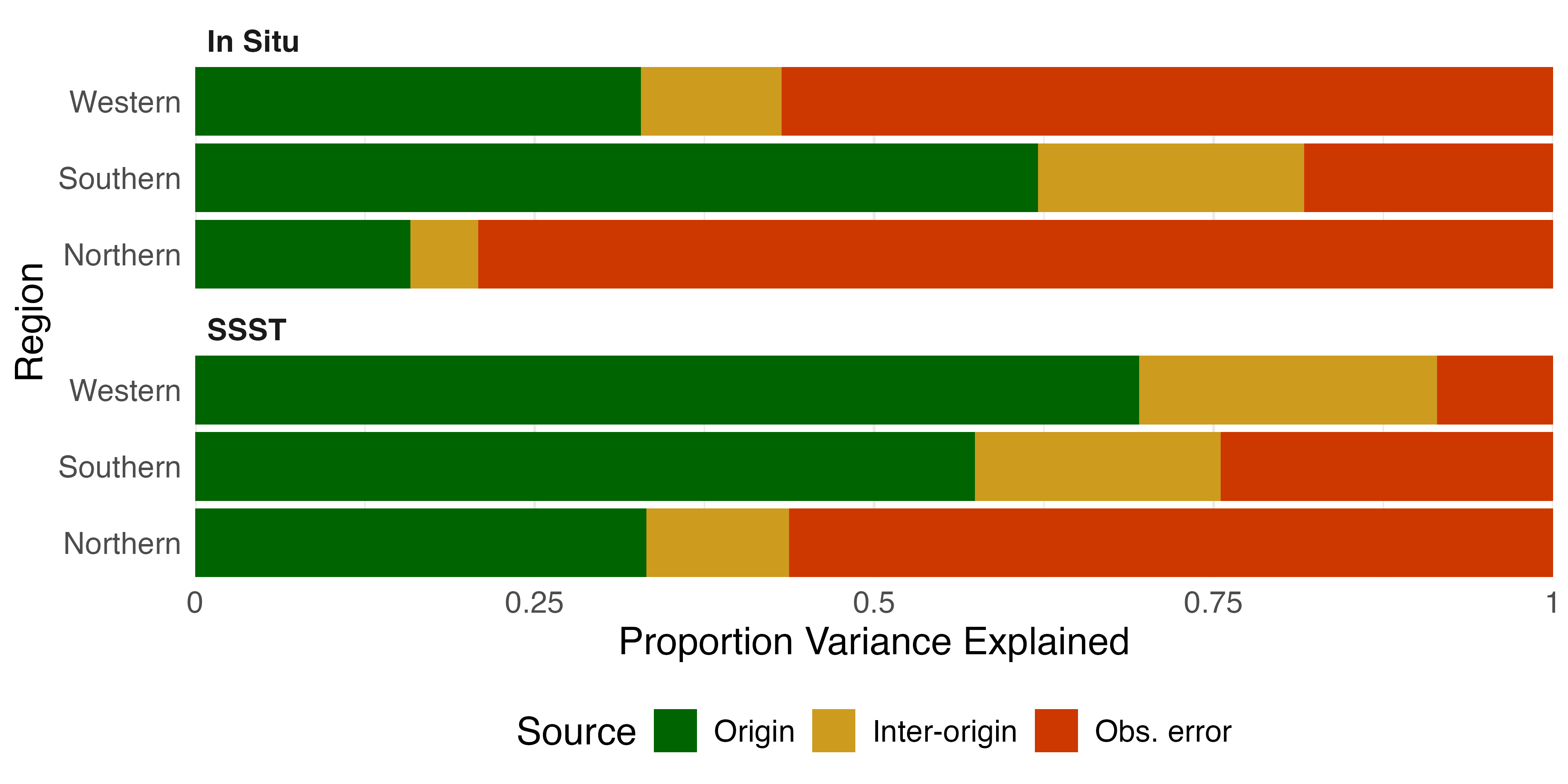

Supplement: S5 Fig — “Origin” refers to variation shared with other regional time series of the same data origin, “Inter-origin” refers to variation which is shared between in situ and SSST data, and “Obs. error” refers to variation specific to individual region- and data origin-specific time series that is not attributable to shared variation with other time series. (TIF) [file pone.0341926.s006.tif]
